# Large scale immersion bath for isothermal testing of lithium-ion cells

**DOI:** 10.1016/j.ohx.2022.e00359

**Published:** 2022-09-20

**Authors:** Mohammad Amin Samieian, Carlos E. Garcia, Laura Bravo Diaz, Alastair Hales, Yatish Patel, Gregory J. Offer

**Affiliations:** a Imperial College London, UK; b University of Bristol, UK

**Keywords:** Immersion cooling, Lithium-ion, Battery, Oil, Temperature control, Thermal management

## Abstract

Testing of lithium-ion batteries depends greatly on accurate temperature control in order to generate reliable experimental data. Reliable data is essential to parameterise and validate battery models, which are essential to speed up and reduce the cost of battery pack design for multiple applications. There are many methods to control the temperature of cells during testing, such as forced air convection, liquid cooling or conduction cooling using cooling plates. Depending on the size and number of cells, conduction cooling can be a complex and costly option. Although easier to implement, forced air cooling is not very effective and can introduce significant errors if used for battery model parametrisation. Existing commercially available immersion baths are not cost effective (∼£3320) and are usually too small to hold even one large pouch cell. Here, we describe an affordable but effective cooling method using immersion cooling. This bath is designed to house eight large lithium-ion pouch cells (300 mm × 350 mm), each immersed in a base oil cooling fluid (150L total volume). The total cost of this setup is only £1670. The rig is constructed using a heater, chilling unit, and a series of pumps. This immersion bath can maintain a temperature within 0.5 °C of the desired set point, it is operational within the temperature range 5–55 °C and has been validated at a temperature range of 25–45 °C.

## Graphical abstract

**Figure:**
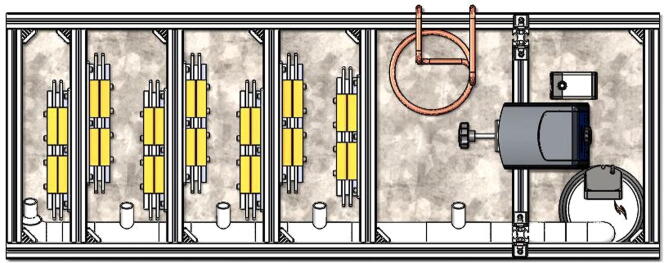

## Specification table

**Table:** 

Hardware name	Immersion cooling rig
Subject area	Engineering
Hardware type	Mechanical engineering and materials science
Open Source License	CC BY 4.0
Cost of Hardware	£1670 (complete breakdown is included in the Bill of Materials in Table 2)
Source File Repository	https://doi.org/10.17632/mjzksgsykm.2

## Hardware in context

The battery industry is expected to increase in size by an order of magnitude or more over the next few decades, as global society shifts towards a low-carbon economy, by electrifying transportation and storing energy from renewables [1]. The increase in demand for batteries with increased energy density has driven the development of lithium-ion cells, with new chemistries and a wide range of form factors [2], [3]. Manufacturers are producing cells for a wide scale of applications, from power electronics such as cordless hand tools, to automotive or grid energy storage with each application having its own power requirements. For example, hybrid electric vehicles require very high-power cells, which can go through multiple charge and discharge cycles over the course of a few minutes, whilst full electric vehicles and personal electronic devices require cells with high energy density which will be discharged over many hours [4].

The performance and lifetime of each cell is unique; it depends on the cell chemistry and the manufacturer [5]. Furthermore, due to the current rate of innovation in the battery industry, a particular cell model will typically become obsolete after 3–5 years. The state of the industry means that there is an enormous amount of battery testing being conducted across the world, in what are most often referred to as cell ‘characterisation’ experiments [6], [7]. Characterisation experiments enable engineers to understand and optimise the use of their chosen cell [8], [9], [10], [31].

Temperature and thermal gradients affect every aspect of lithium-ion cell operation and performance. For example, a cell will have a higher impedance at lower temperatures and be generally less capable of passing current in either charge or discharge [11], [12]. Furthermore, at both high (>45 °C) and low (<15 °C) temperature extremes, the cell will rapidly degrade [13], [14]. Thermal gradients add an extra layer of complexity; thermal gradients within a cell lead to impedance inhomogeneity, which in turn causes different regions of the cell to perform differently as electrical current is non-uniformly generated throughout the cell. Such thermal gradients have been shown to cause accelerated degradation of the cell [11]. The contribution of these thermal gradients towards degradation can sometimes be larger than the effect of higher average absolute temperatures [6]. In reality, it is not always possible to perfectly control the temperature across every cell in a battery pack, but it is essential that a cell’s temperature-dependent performance is properly understood to enable studies of the effect of temperature variation across a battery pack [15]. This is done by developing temperature dependent models, but these require experimental data from testing cells with accurate temperature control. As a result, it is widely established within the battery testing community that temperature control during cell testing is crucial.

Heat generation occurs inside every lithium-ion cell during testing which complicates temperature control during characterisation experiments [6], [16], [17]. Cell heat generation during experimentations is combated by incorporation of a thermal management system into the experimental setup. Climate chambers, employing forced convection cooling, are currently the dominant solution throughout the battery community [18], [19], [20]. They induce heat transfer from the cell under test by blowing air, using fans, across the exposed surfaces. Climate chambers are fitted with heaters and refrigeration units to accommodate high and sub-ambient temperature testing. They are popular because they allow very easy access for the experimentalist and are reasonably space effective in a test facility. However, climate chambers are unable to effectively control the temperature of a lithium-ion cell that is generating its own heat. Air is not a sufficient working fluid for the rates of heat transfer required, and the cell, under test, will inevitably heat up over the course of the experiment [21]. In a recent study conducted in a climate chamber, Cavalheiro *et al.* observed a cell surface temperature rise of 14 °C during a routine characterisation experiment [22]. This is a major problem because the temperature rise affects the cell’s characteristics and subsequent operation. The experimentalist is left with an additional undesirable variable in their results, which affects every other cell characteristic they were aiming to quantify. The problem intensifies as the energy density of cells increase, or at higher currents, as this results in higher heat generation per unit volume.

Conduction cooling is an attractive alternative [12], [23], [24]. Typically, cooling plates are attached to a cell’s surfaces and their temperature is controlled through Peltier elements. Such apparatus is highly functional, for example Ardani *et al.* reduced temperature variation by >80 %, compared to equivalent tests conducted in a climate chamber [7]. The problem of heat removal, however, still remains; heat must be removed from any such conductive cooling device and this adds significant complexity to the design. Critically, conductive cooling apparatus are unable to accommodate a range of different cells. Most often, a bespoke apparatus is designed and manufactured for an individual cell model and a single experiment. The solution is not economically justifiable for a large proportion of the battery industry.

Another alternative method is immersion cooling. Immersion cooling is an effective way to control the temperature of a large number of cells. This is an attractive method because it is better than air cooling and does not have the complications associated with conduction cooling. In addition, this setup is far less geometry dependant and a range of different cells can be cooled.

In this paper, we introduce the design of a cost efficient, large immersion bath specifically designed for cell testing. Each bath can accommodate at least eight large format pouch cells, however, the apparatus is easily adaptable to accommodate multiple cell sizes and form factors. The design incorporates temperature control between 5 °C and 55 °C. The dielectric immersion fluid can achieve three orders of magnitude higher rate of convective heat transfer than may be achieved with air.

## Hardware description

We had five requirements for our immersion bath, as listed below:

• The capability to both chill and heat the immersion fluid;
• Precision temperature control of the immersion fluid to within ±0.5 °C;
• Simple and robust temperature control system;
• Sized to accommodate eight large lithium-ion pouch cells (>50 Ah);
• Low cost.

Immersion baths available on the market target single cell testing applications or are borrowed from other research fields. Bath sizes are typically an order of magnitude smaller than our requirement. This immersion bath design meets the five requirements at a fraction of the cost of the smaller immersion baths in the market and is based on components that can easily be sourced.

**Fig. 1:**
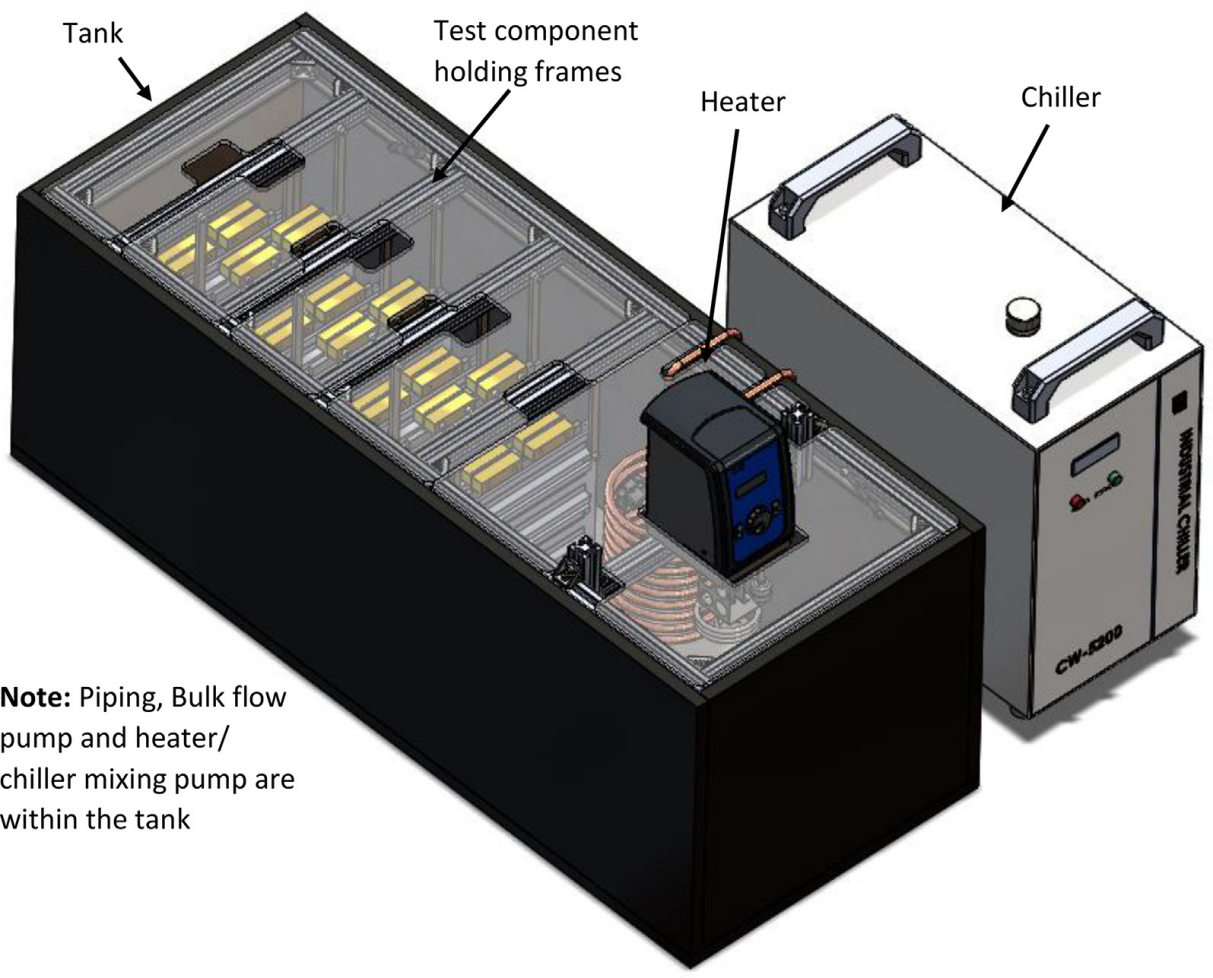
Immersion bath assembly.

The immersion bath is made of six main components (Fig. 1): tank, test component holding frames, the heater, the chiller, the bulk flow pump and the heater/chiller mixing pump. In addition, the piping topology is arranged to ensure thorough mixing throughout the volume of the immersion bath, and thus to maintain a uniform temperature.

The heater and chiller work alongside each other to achieve the required temperature without a complex control system. The heater is set to the desired temperature, which it can achieve with an accuracy of ±0.1 °C [25]. Heat is added to the immersion bath at a higher rate than the chiller can remove heat. The chiller is used for temperature stabilisation, providing a near constant rate of heat removal to reduce the overshoots achieved by the heater. This is achieved by setting the chiller to a temperature below that of the heater. The immersion bath will therefore settle at the temperature selected on the heater (see Table 3 for further details).

## Design files

The design files for the immersion bath can be found in Table 1.

**Table 1:** Design file summary.

Design file name	File type	Open source license	Location of the file
Immersion bath CAD	CAD	CC BY 4.0	https://doi.org/10.17632/mjzksgsykm.1

### Bill of materials

The bill of materials for making of one unit of the immersion bath is shown in Table 2.

**Table 2:** Bill of materials for making one unit of the Immersion bath. *NB: oil was supplied by the Petronas Lubricants International at no cost for evaluation purposes. Any dielectric oil may be used at estimated cost £2/L. Please note the ideal oil/fluid that is to be used for cooling batteries should be dielectric and non-hygroscopic.

Item	Manufacturer & Part number	Description	Qty	Total cost/ £
Aluminium extruded profile 30 × 30 mm	KJN [26]	1.2 m	8	75.50
Brass blocks	Local suppliers	C121 brass	28	91.60
Chiller	B&S CW-5200 [27]	–	1	337.67
Copper immersion coil	Local suppliers	8 m × 135 mm dia.	1	30.67
Galvanised trough	Local suppliers	1219 × 457 × 405 mm	1	88.00
Submersible pump	Mizar 30/S [28]	–	1	188.00
Heater	Grant T100t [25]	1.2 kW heater	1	604.97
Mending plates	Local suppliers	Mending plates zinc-plated 76 × 16 × 10 pack	8	1.79
Angle brackets including fasteners	Bosch	30 mm, 8 mm slot	20	34.81
Insulation	Armaflex class O [29]	AF Sheets 200 cm × 50 cm	4	151.01
Submersible Water Pump	Powcan	400GPH 1500L/H	1	20.99
Base Oil	–	150 L	1	n/a*
pipe	Davant [30]	3 m – 2″diameter	1	8.78
pipe elbow	Davant [30]	2″ – 90°	4	9.68
pipe *T*-connector	Davant [30]	2″	4	6.52
Pipe reducer	Davant [30]	2″ to 1.25″	5	17.00
PVC pipe cement	Everbuild P16	–	1	4.49

#### How temperature control is achieved

**Fig. 2:**
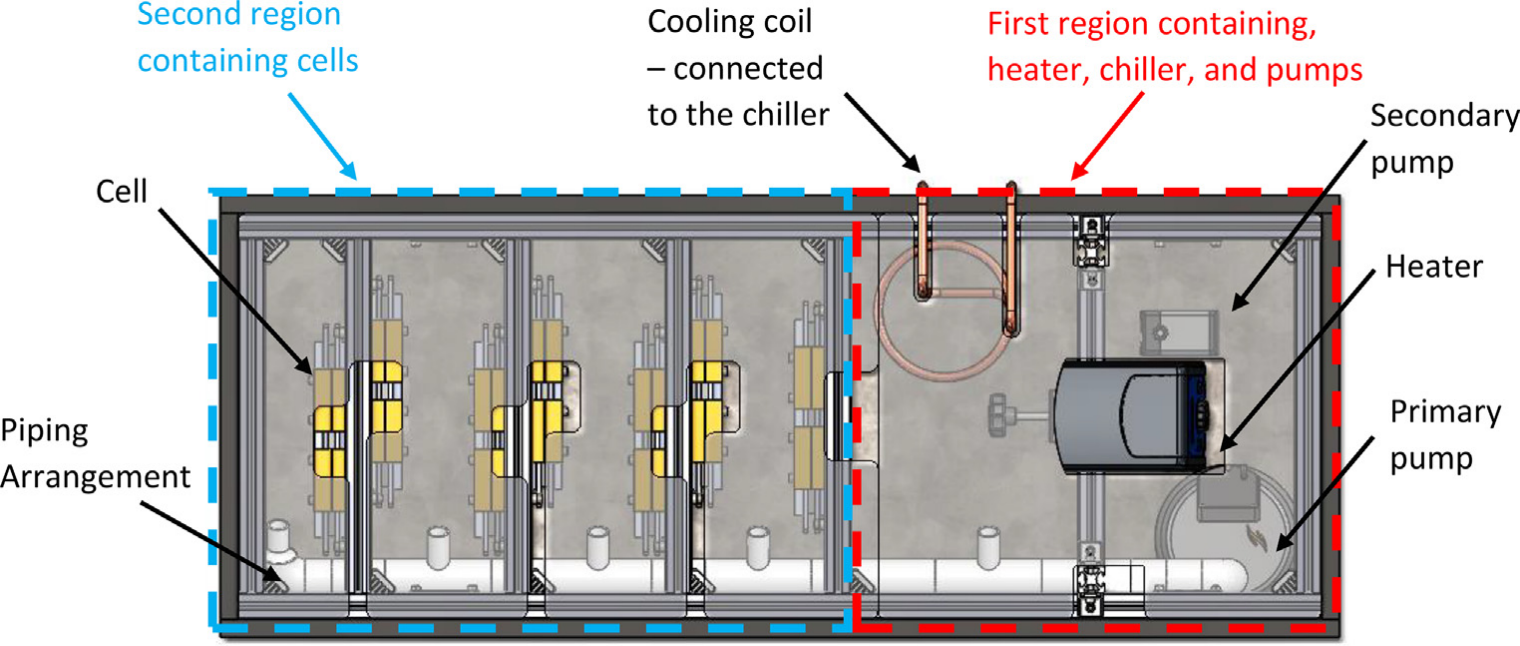
Top view of the bath showing two regions, and pump and piping arrangements.

Cells and other components are arranged in the bath in two different regions (Fig. 2). The first region houses the heater, chiller and pumps with the second region housing the cells. However, no physical barrier separates these regions. It is important to ensure that the heater switches off once a uniform temperature is reached across both regions of the bath. This can only happen if the temperature probe in the heater is able to read an accurate temperature representative of both regions of bath. This has been achieved by having two pumps. The primary pump ensures uniform mixing of the oil between the two main regions of the bath. The secondary pump is near the chilling and heating elements to ensure good mixing locally.

The primary pump was used to achieve good mixing between the two regions of the bath. By carefully laying out the piping arrangement, oil was pumped to each location where cells were mounted to ensure uniform flow across every cell and hence uniform temperature. The secondary pump was used to allow for good mixing in the proximity of the heating and chilling elements in the oil. This was important for two reasons. The first was that local heating or chilling does not directly affect the temperature probe in the heater. The second reason was that a uniform mixing of the oil was achieved locally at the intake of the primary pump.

Temperature stability was one of the key requirements in the design of this apparatus and this was achieved by allowing the heater and chiller to work alongside each other. This was because the chiller counterbalances the unwanted temperature rise if an internal or external heat source other than the heater was to inject heat into the bath. Two main internal heat sources are present, the heat generated by the cells during testing and the heat from the pumps during operation. This is in addition to the variability of the external environmental temperature.

In use, the heater switches off automatically when the set temperature is reached. However, significant heat is produced from the pump and dissipated into the bath even when the heater is idle. The chiller, set to cool the bath at a constant rate throughout testing, counterbalances this heating effect. During periods where the chiller is removing excessive heat, the heater is automatically triggered into operation. In this manner, the bath temperature is maintained by the heater’s intermittent operation, without needing a combined control system for the heater and chiller together.

## Build instructions

### The bath container, its frame and insulation

1. Drill four holes at locations shown in Fig. 3 on the edge of the tank.

**Fig. 3:**
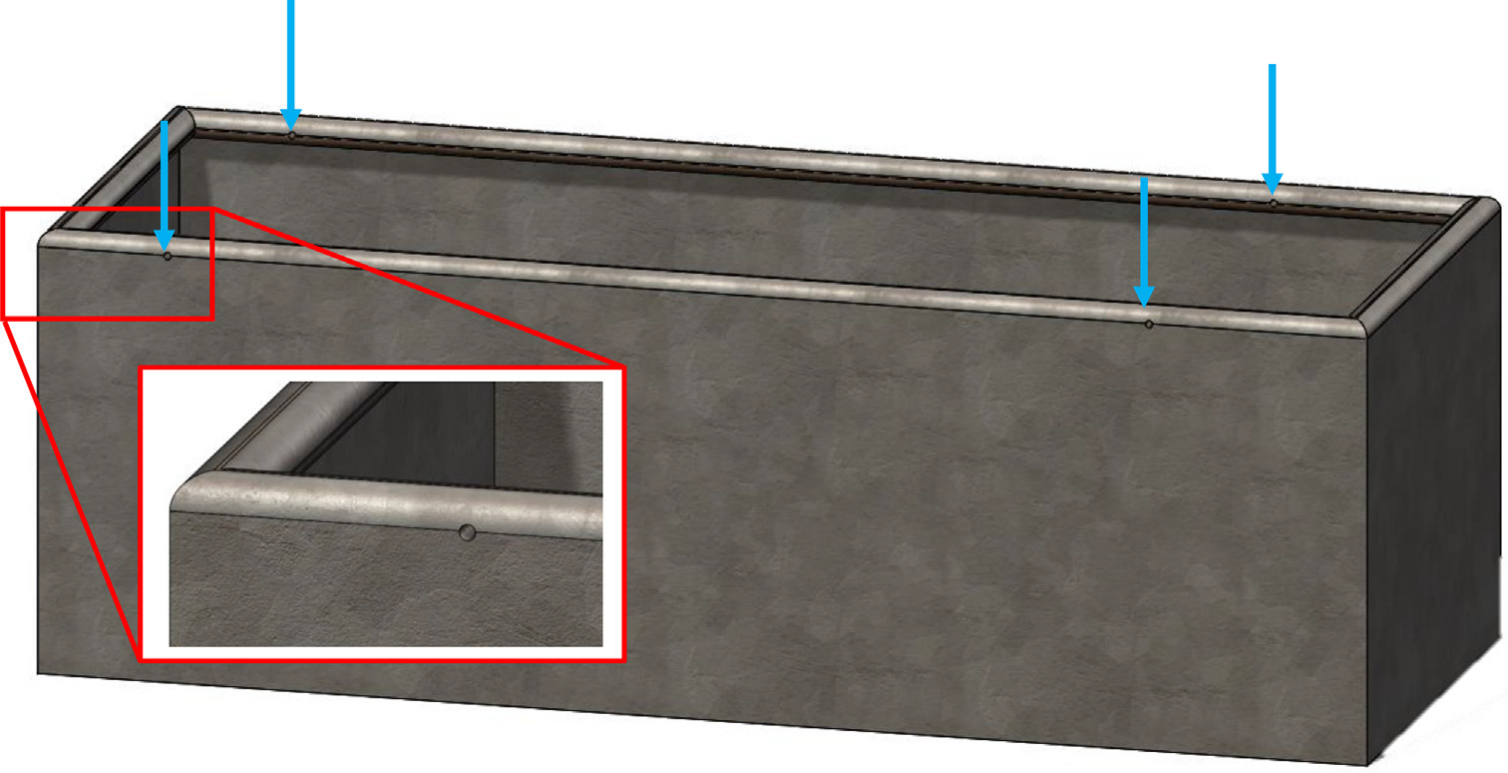
Location of the four holes on the tank (shown in blue arrows) required for attaching the main frame to top of the tank. (For interpretation of the references to colour in this figure legend, the reader is referred to the web version of this article.)
2. Build a rectangular frame using aluminum extrusions and brackets. This frame will be referred to as the main frame in the later stages of the assembly.
3. It is important to add the insulation to all sides of the tank in the early stages of the assembly to ease the process. This is particularly important for the bottom of the tank. The insulation can be cut to the dimensions required, then attached to the sides of the tank by applying adhesive.
4. Attach rectangular frame to the top of the tank using mending plates (Fig. 4).

**Fig. 4:**
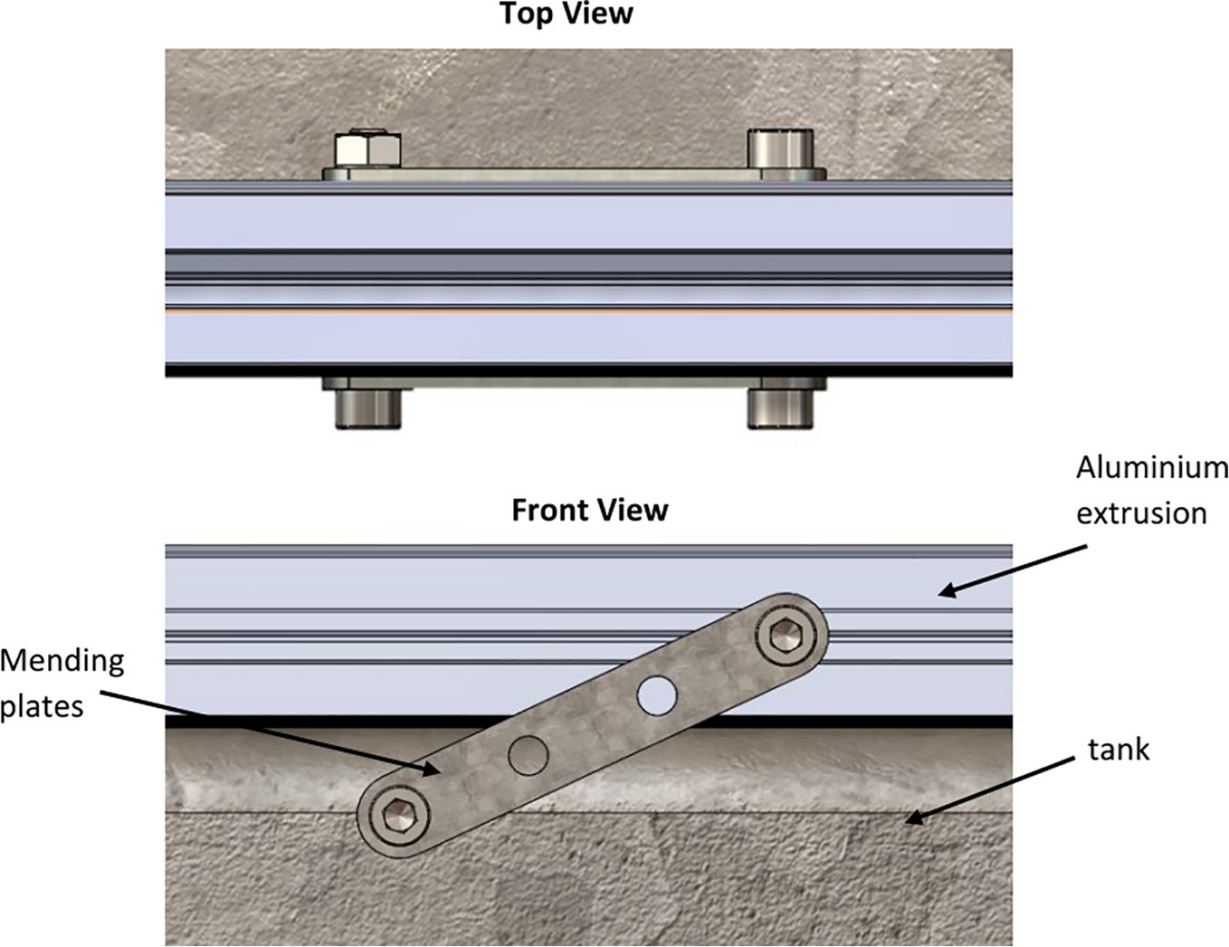
Connection of the main frame to the tank using mending plates.

### Pump and piping

**Fig. 5:**
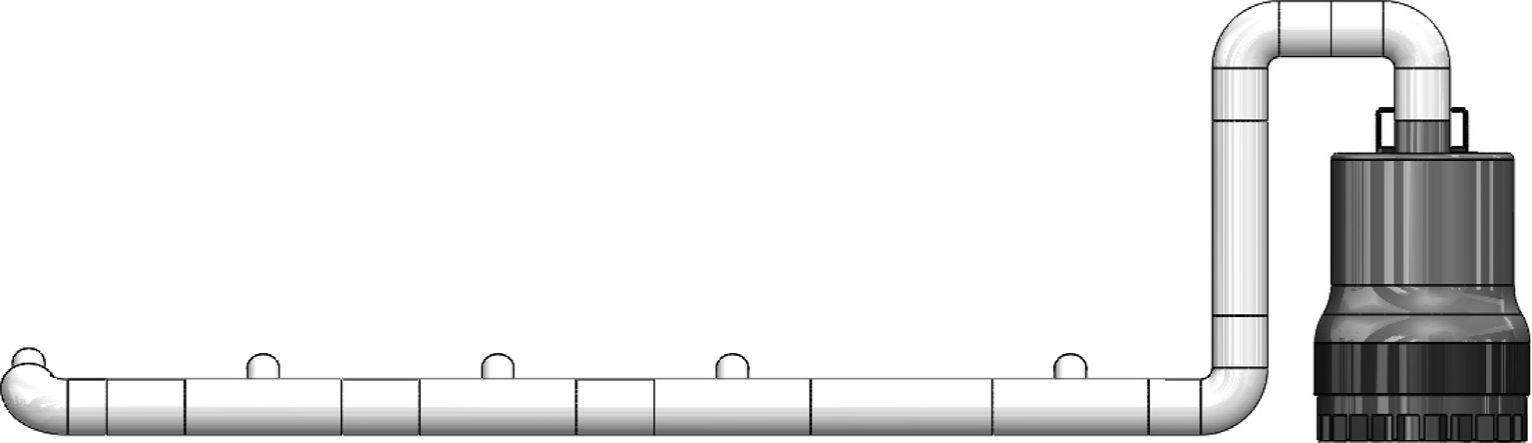
Primary pump along with its piping assembly. This is the view in which it was placed withing the length of the tank.
4- Apply PVC pipe cement to secure the pipe connections.
5- Place secondary pump in the desired location (see Fig. 2).

1- Take measurements across the length of the tank from the primary pump for the desired exit locations of the oil at the different cell locations. Then cut pipe to dimension.
2- Pipe elbows, *t*-connectors, and reducers should then be connected to the desired locations measured in the previous step. Dry-fit (without applying Poly-Vinyl Chloride (PVC) pipe cement) at this stage to allow for changes that may need to be made.
3- Assemble piping arrangement and primary pump in the tank to ensure it is in the correct location and make any necessary adjustments (Fig. 5).

### Heater and chiller

1- The aluminum extrusion bar to which the heater will be attached to, should be attached to the main frame using angle brackets.
2- The heater should then be attached to the bar.
3- The chiller should be attached to a copper coil using flexible pipes. Care should be taken such that the water in the loop of the chiller does not mix with the oil.
4- The copper coil can then be hung from the edge of the tank in its allocated location.

### Cells

**Fig. 6:**
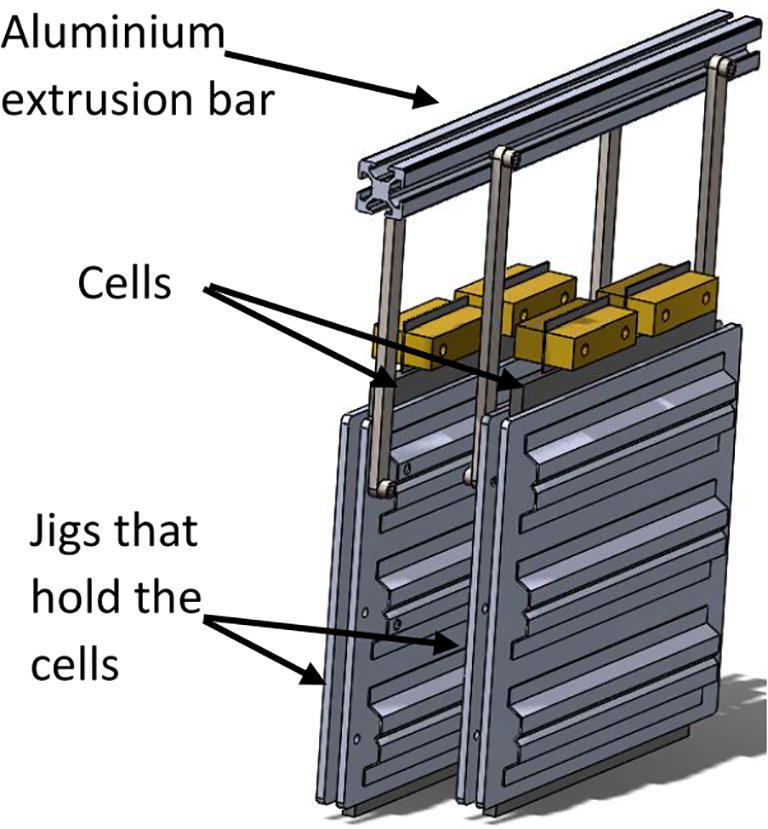
Two cells mounted in jigs and connected to an aluminium extrusion bar.
2- Two jigs should be attached to an aluminum extrusion bar prior to the bar being attached to the main rectangular frame.
3- The bars can be attached one by one using angle brackets to the main rectangular frame.
4- In our implementation, 56 Ah lithium-ion pouch cells with NMC chemistry are used.

1- Each cell is mounted on a jig – provided by the cell supplier (Fig. 6).

### Fill bath

At this stage, the bath should be filled with oil or other desired fluid depending on the application. In applications where electrical conductivity is not an issue, de-ionised water could be used, along with inhibitor and biocide to prevent mould growth.

### Cover

1- The cover was laser cut into two sections to allow for easier assembly. Profiles were laser cut on the cover to allow for the cables from the cells to pass through. Depending on the application that this immersion bath will be used for, the lid can be further split into smaller sections to allow an easier assembly process.
2- A layer of insulation should then be placed on top of the cover to minimize heat exchange between the fluid within the bath and the environment.

### Operation instructions

1- Turn pumps on
2- Turn chiller on
3- Turn heater on
4- Use Table 3 as a guide for setting the temperatures on the chiller and heater depending on a required bath temperature

**Table 3:** Temperature setting guide for heater and chiller.

Required bath temperature	Set heater temperature to:	Set chiller temperature to:
25 °C	25 °C	5 °C
35 °C	35 °C	20 °C
45 °C	45 °C	35 °C

### Validation

It was essential that the immersion bath design facilitated effective and uniform cooling across the jigs that hold the cells. For this reason, as well as ensuring thorough mixing of the immersion fluid, it was important to achieve consistent rates of heat transfer from both surfaces of every jig. This would prevent the build-up of a temperature difference from one surface of a jig to the other.

Two sets of tests were performed to confirm that the requirements of thorough bulk coolant mixing, and uniform jig cooling were both met. Preliminary tests were performed with water, and actual validation was carried out using oil. The advantage of performing preliminary tests with water first was that it was easier to drain and make any necessary modifications than it would be once the tank is filled with oil.

**Fig. 7:**
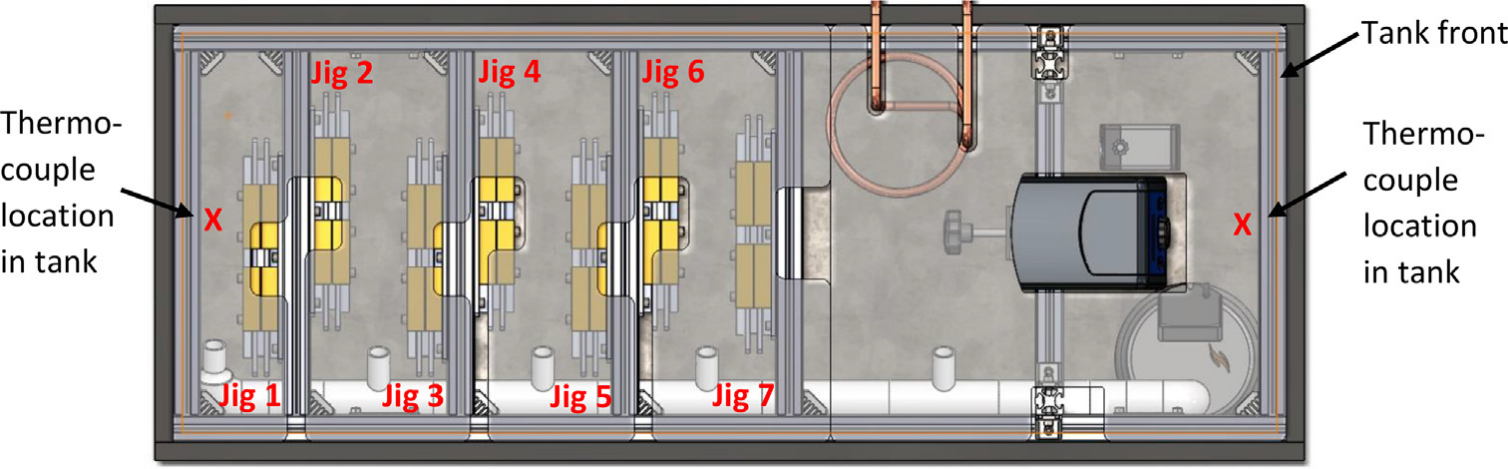
Cell/jig locations are numbered from one to seven in order of from left to right. The front of the tank is also labelled, as it is referenced in the thermocouple location, temperature data. Two thermocouples spaced 20 cm apart in depth, were placed at each of the two locations in the tank shown with an X and also a thermocouple was placed at the centre of every jig.

During both the water tests and the oil tests, the temperature of the cells (or jigs that hold the cells) was measured using K-type thermocouples mounted on the surface at the centre of each jig. Pico Technologies TC-08 units were used for temperature logging. The location where the cells were mounted in shown in Fig. 7. Please note, the accuracy of K-type thermocouples is typically ±1.5 °C over the range –40 to +1000 °C.

## List of symbols used in the validation section

The symbols used throughout the validation section are defined in Table 4.

**Table 4:** List of symbols used.

*T*_initial_	Temperature of the fluid at the start of the experiment
*T*_final_	Temperature of the fluid at the end of the experiment
∂*T*/∂*t*	Rate of temperature change
*ΔT*_between jigs_	Temperature difference between jigs
*ΔT*_jig sides_	Temperature difference between front and back side of each jig
*T*	Instantaneous temperature of the cooling fluid
*t*	Duration the temperature was kept constant
*ΔE*	Thermal energy added to the tank during thermal perturbation test
*t*_mixing_	Time taken for the fluid to return to an isothermal condition after experiencing a forced thermal perturbation
*ΔT*_15 s_	Maximum temperature difference between jigs after 15 s of a forced thermal perturbation
*ΔT*_120 s_	Maximum temperature difference between the jigs after 120 s of a forced thermal perturbation
*ΔT*_between tank top and bottom_	Temperature difference between top and bottom of the tank
*ΔT*_tank front and back_	Temperature difference between front and back of the tank
*ΔT*_cells_	Temperature difference between different cells

### Preliminary water tests

A first batch of tests is performed using water, to ensure the rig is working satisfactorily before adding oil because adding oil to the rig too early would complicate any subsequent modifications or adjustments. The purpose of these tests is twofold: first, to test the overall behaviour of the immersion bath including the heater, chiller, pump and thermocouples; and second, to test the effectiveness by monitoring any temperature imbalances between the compression jig surfaces. Five water tests were conducted without the presence of the cells to avoid short circuiting the cells.

**Fig. 8:**
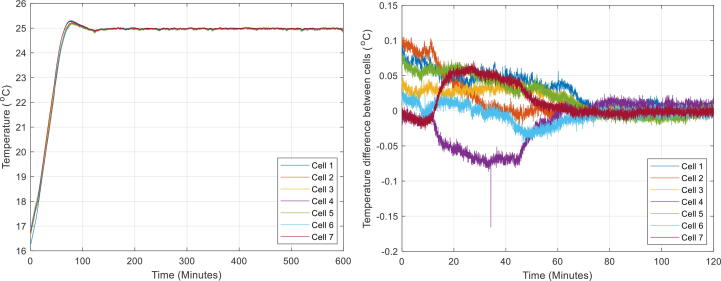
Temperature from the first test whilst bath was heated to 25 °C (left) and maximum difference between the different cells in the initial stage of heating up to a temperature of 25 °C (right).

The first and second test looked at investigating temperature homogeneity between the seven cells, mounted into the seven jigs in the immersion tank (Table 5). The first test heated the bath to 25 °C from an initial temperature of 16 °C (Fig. 8). The second test heated the bath to 35 °C from an initial temperature of 25 °C. The difference in average cell temperature was found to be small (<0.5 °C). The temperature gradient across each jig was also very low (<0.1 °C), therefore, it is safe to assume the immersion fluid is thoroughly mixed throughout testing. In addition, during the heating process, the temperature for each jig followed the same path and reached the setpoint at a very similar time, therefore it is assumed that the heating/cooling rate at each surface of the compression jig is equal.

**Table 5:** Summary of heating test results.

Test #	*T*_initial_ (°C)	*T*_final_ (°C)	∂*T*/∂*t* (°C/h)	*ΔT*_between jigs_ (°C)	*ΔT*_jig sides_ (°C)
1	16	25	8.0	<0.5	<0.1
2	25	35	6.7	<0.5	<0.1

The third and fourth test looked at temperature stability (Table 6). Both parts looked at temperature stabilisation over ten hours, the first part was done at 25 °C (see Fig. 8 left) and the second part at 35 °C. The results showed that the temperature control system can maintain a very consistent bath temperature, with fluctuations of < 0.1 °C.

**Table 6:** Results summary of thermal stability tests.

Test #	*T* (°C)	*t* (h)	*ΔT*_jig sides_ (°C)
3	25	10	<0.03
4	35	10	<0.05

**Fig. 9:**
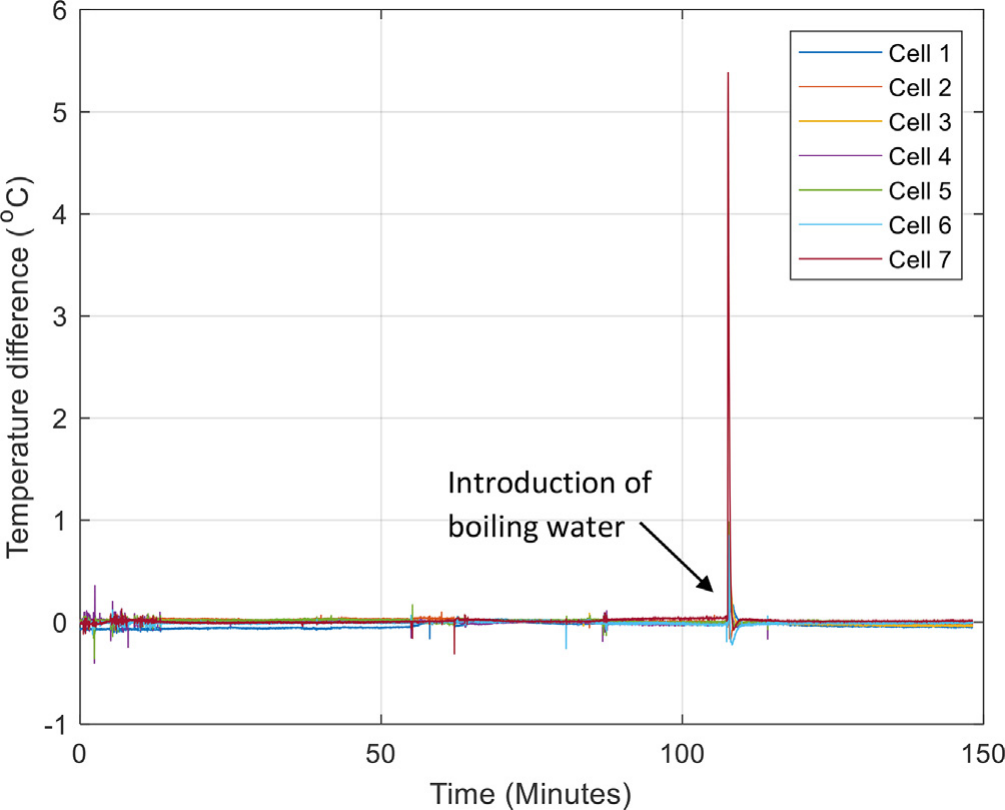
Temperature gradient across each jig during the thermal perturbation test of 250 kJ (fifth test). This was where boling water was added for a duration of five seconds and temperature of the jibs monitored.

The fifth test analysed the response of the system to a thermal perturbation of 250 kJ, added at the site of jig location one over a period of five seconds in the form of boiling water (Table 7). The equilibrium time after which the water had fully mixed was determined, as well as the maximum temperature difference between jigs at 15 s and 120 s after the addition of hot water. The results showed that after 132 s the fluid had returned to thermal equilibrium after being exposed to a sudden thermal imbalance (Fig. 9). The time for the temperature gradient across each cell jig to drop below 0.5 °C was just 15 s. This demonstrates that the recirculation system is very effective at mixing the immersion fluid.

**Table 7:** Results of the thermal perturbation tests. The temperature differences are also shown at 15 s and 120 s after heat addition.

Test #	*ΔE* (kJ)	*t*_mixing_ (s)	*ΔT*_15 s_ (°C)	*ΔT*_120 s_ (°C)
*5*	*250*	*132*	*<0.5*	*<0.05*

This apparatus was designed to be used for oil. All tests so far in this section were performed with water as the immersion fluid. However, oil and water have different fluid properties. It is well established that base oil and water have different thermal properties and therefore are expected to perform differently as immersion fluids for lithium-ion cells.

### Oil tests with cells at rest

**Fig. 10:**
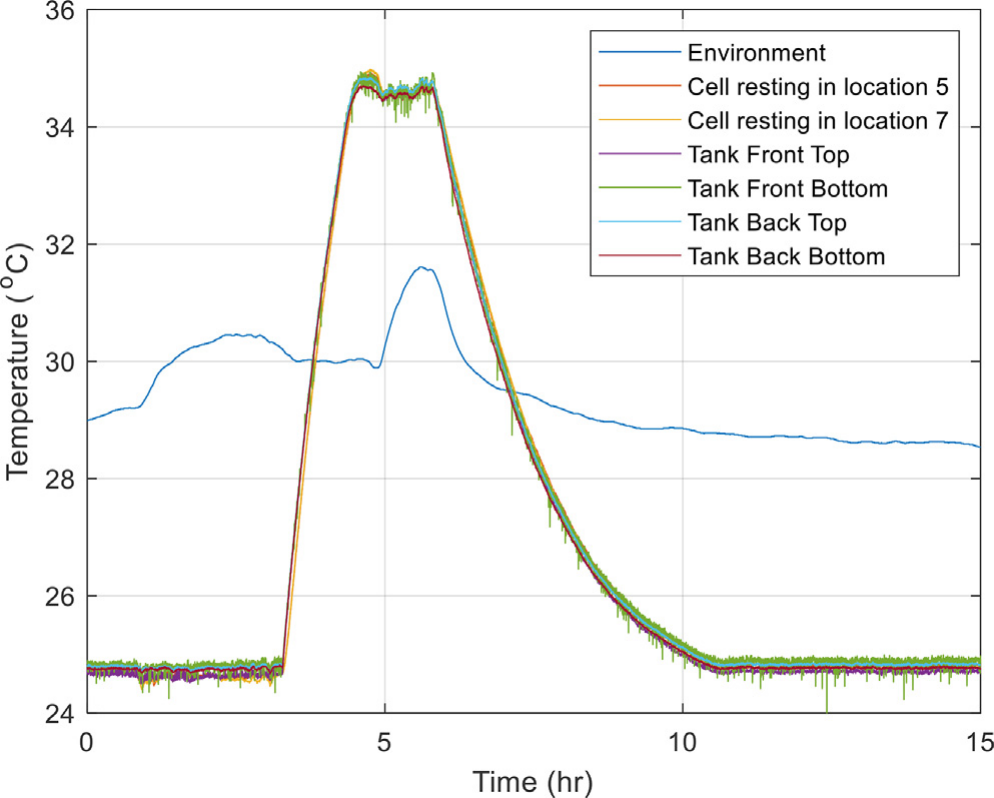
Heating and cooling assessment of the immersion bath. Temperature raised to 35 °C from 25 °C and then back to 25 °C again. Tests done with jigs submerged in oil without the cells being electrically tested.

**Fig. 11:**
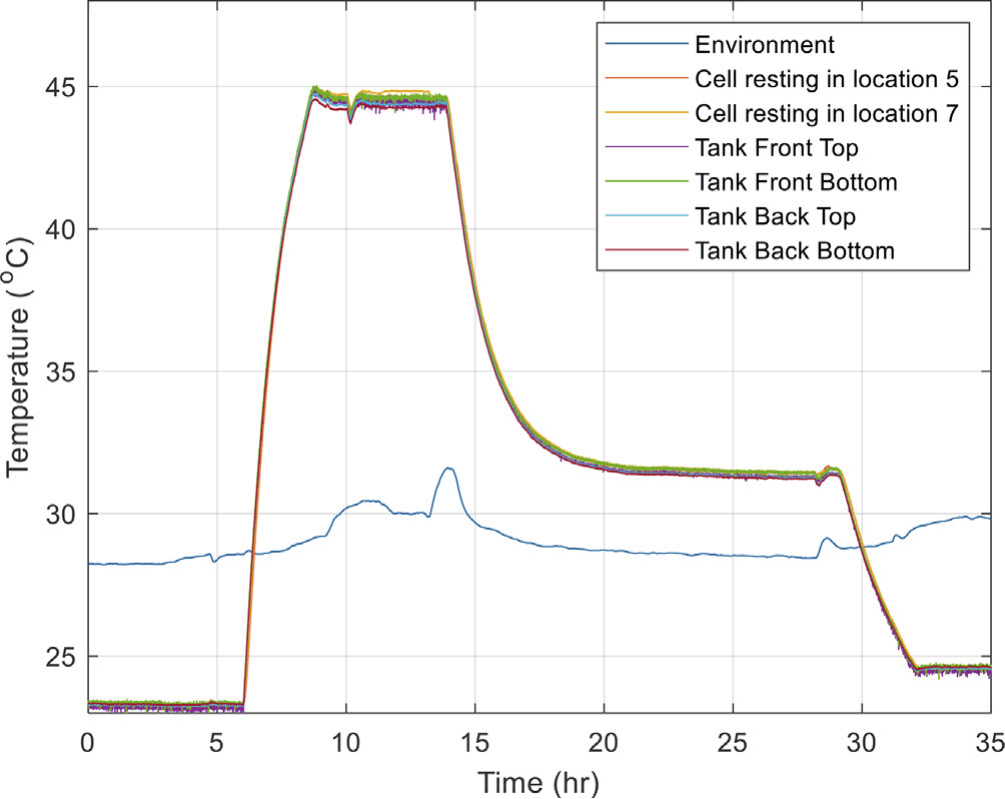
Heating and cooling assessment of the immersion bath up to 45 °C using oil without the cells being electrically tested.

The purpose of these experiments was to assess the overall functioning of the immersion bath and to measure the time taken to reach a stable temperature when using oil. The temperature of the bath was both increased and decreased so that the time taken in both scenarios could be measured. Temperature across different locations in the bath and on the surface of two of the cells in resting were recorded. The results are summarised in Table 8 and Table 9. The temperature measurements from both different experiments are shown in Fig. 10 and Fig. 11.

**Table 8:** Heating and cooling assessment up to 35 °C using oil. The cells were placed in locations 5 and 7. The thermocouples in the tank are located in opposite corners front and back of the tank (Fig. 7) with two thermocouples in each location, one on the top and one at the bottom of the tank.

Test #	*T*_initial_ (°C)	*T*_final_ (°C)	∂*T*/∂*t* (°C/h)	*ΔT*_between tank top and bottom_ (°C)	*ΔT*_tank front and back_ (°C)	*ΔT*_cells_ (°C)
1	25	35	7.0	<0.2	<0.4	<0.1
2	35	25	2.1	<0.2	<0.4	<0.1

**Table 9:** Heating and cooling assessment up to 45 °C using oil. The cells were placed in locations 5 and 7. The thermocouples in the tank are in opposite corners front and back of the tank (Fig. 7) with two thermocouples in each location, one on the top and one at the bottom of the tank.

Test #	*T*_initial_ (°C)	*T*_final_ (°C)	∂*T*/∂*t* (°C/h)	*ΔT*_tank top and bottom_ (°C)	*ΔT*_tank front and back_ (°C)	*ΔT***_cells_** (°C)
1	23	45	7.9	<0.2	<0.4	<0.1
2	45	32	2.0	<0.2	<0.4	<0.1
3	32	25	2.3	<0.2	<0.4	<0.1

### Oil tests while cells undergoing constant current cycling

An experiment was also carried out while the cell was being electrochemically charged and discharged at constant current (CC). The aim of this experiment was to observe any variation in tank temperature as a result of the cells heating up during electrochemical cycling. One of the cells was cycled at a moderate C-rate^1^ (1C – taking one hour to charge the cell) and mounted in jig 5. The other cell was cycled at a relatively high rate (3C – taking a third of an hour to charge the cell) and mounted in jig 7.

**Fig. 12:**
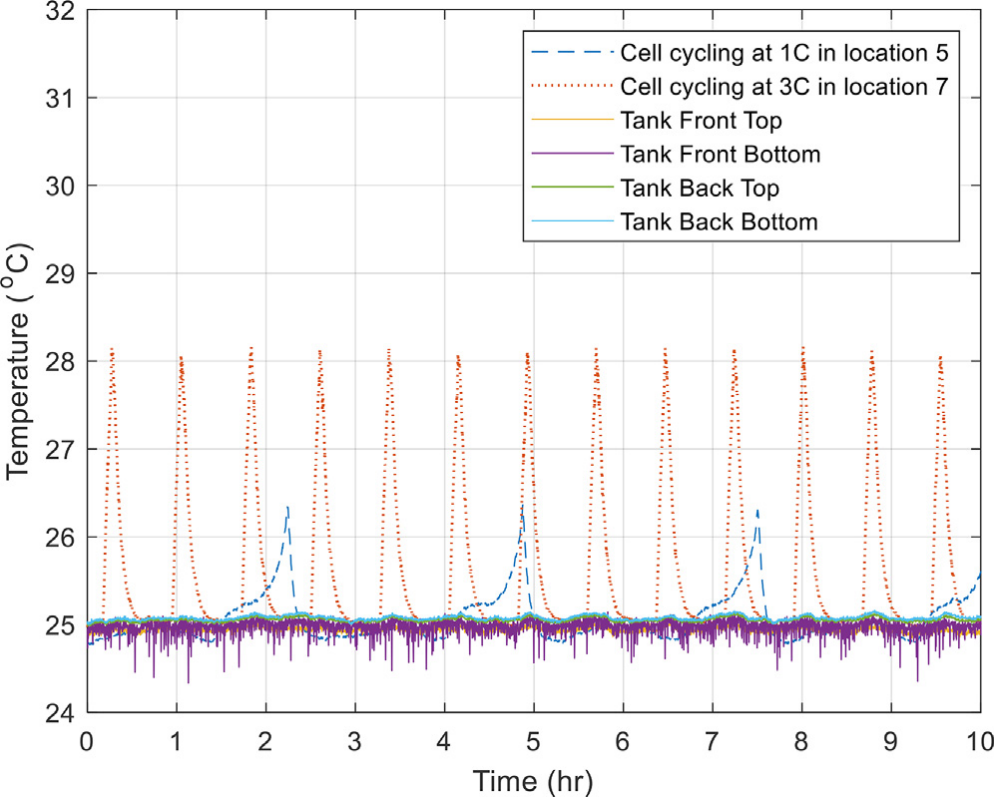
Temperature variation across the tank while the cells were electrically tested at 25 °C.

**Fig. 13:**
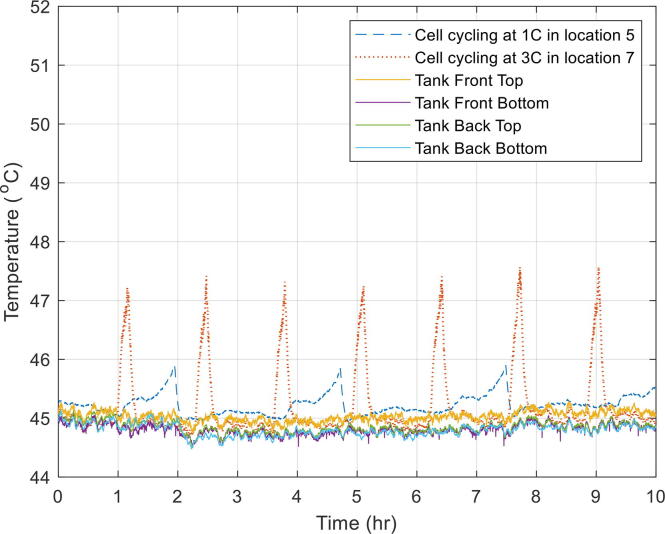
Temperature variation across the tank while the cells were electrically tested at 45 °C.

This experiment was carried out at a tank temperature of 25 °C (Fig. 12) and at 45 °C (Fig. 13). The results showed that despite the cells surface temperature rising, the impact on overall tank temperature was almost negligible.

## Conclusions

We have presented a low-cost, robust temperature control system for immersion cooling of large lithium-ion pouch cells, capable of controlling the temperature of the bath to within 0.5 °C. Despite its large size (150 L), the design presents efficient fluid mixing, leading to homogeneous temperatures across the bath. Besides the thermal management of large lithium-ion batteries, this design is also suitable for the thermal management of supercapacitors, lithium-hybrid capacitors and any other type of large batteries, electrical components, or even non-electrical components. The oil used in the tank may be replaced with other fluids depending on the application and/or budget constraints.

## Declaration of Competing Interest

The authors declare that they have no known competing financial interests or personal relationships that could have appeared to influence the work reported in this paper.

## Footnotes

1 C-rate is defined as the rate of charge of discharge, expressed in terms of the nominal capacity of the cell. For example, during a 2C discharge the cell becomes fully discharged after ½ hour approximately.
